# Development and Role in Therapy of Canakinumab in Adult-Onset Still’s Disease

**DOI:** 10.3389/fphar.2018.01074

**Published:** 2018-09-21

**Authors:** Paola Galozzi, Chiara Baggio, Sara Bindoli, Francesca Oliviero, Paolo Sfriso

**Affiliations:** Rheumatology Unit, Department of Medicine DIMED, University of Padova, Padova, Italy

**Keywords:** Canakinumab, Adult-onset Still’s disease, Interleukin-1 beta, drug development, therapy

## Abstract

Adult-onset Still’s disease (AOSD) is a rare inflammatory disease of unknown etiology typically characterized by episodes of spiking fever, evanescent rash, arthralgia, leukocytosis, and hyperferritinemia. The pivotal role of interleukin (IL)-1 and other pro-inflammatory cytokines gives rise to the development of new targeted therapies. Currently, AOSD patients can benefit from efficient and well tolerated biologic agents, such as IL-1, IL-6, and tumour necrosis factor (TNF)-α antagonists. Canakinumab, a human monoclonal anti-IL-1β antibody, is indicated for the treatment of different autoinflammatory syndromes in adults, adolescents, and children and it has recently been approved for AOSD treatment. In this article, we summarize the structural and biochemical data describing the molecular interactions between Canakinumab and its target antigen. Some special considerations of the pharmacological properties of Canakinumab are included. We also review the safety, efficacy and tolerability of this drug for the treatment of AOSD.

## Introduction

Adult onset Still’s disease (AOSD) is a rare autoinflammatory disorder of unknown etiology characterized by high spiking fever, salmon-like evanescent rash, and joint involvement. Other symptoms are usually reported in AOSD, such as pharyngitis, myalgia, splenomegalia, hepatitis, and abdominal pain (Kadavath and Efthimiou, 2015; Sfriso et al., 2016). The disease is very heterogeneous, ranging from benign forms to severe life-threatening complications, such as macrophage activation syndrome (MAS). Also the laboratory findings are highly unspecific. High levels of C-reactive protein (CRP), increased erythrocyte sedimentation rate (ESR), blood neutrophilia, hyperferritinemia, and elevated liver enzymes.

Adult-onset Still’s disease is a very uncommon disease, with an annual incidence estimated between 0.16 and 0.62 per 100,000 persons worldwide, independent of ethnic group (Magadur-Joly et al., 1995; Wakai et al., 1997; Evensen and Nossent, 2006; Balci et al., 2015). Recent epidemiologic data from the Northwestern Thrace Region in Turkey reported an overall prevalence of 6.77/100,000 between 2003 and 2014 (Balci et al., 2015), consistent with Norwegian point prevalence data of 6.9/100,000 in 2000 (Evensen and Nossent, 2006). A Japanese nationwide survey reported, on the other hand, an estimated prevalence of 3.9/100,000 in 2010/2011 (Asanuma et al., 2015).

In light of new evidence, a dichotomous classification distinguishes two AOSD subtypes according to clinical evolution: a systemic subtype, including patients with systemic features and more at risk to develop life-threatening complications, and a subtype where patients have predominantly articular involvement (Maria et al., 2014).

Adult-onset Still’s disease shares common clinical and biological features with another systemic inflammatory condition, called systemic onset Juvenile Idiopathic Arthritis (SoJIA), affecting children aged 16 years or younger (Jamilloux et al., 2015). This supports the concept of a Still’s disease continuum that includes both the juvenile onset (SoJIA) and adult onset (AOSD) form. Evidence also suggests that both diseases are comparable at the molecular level, probably due to the activation of similar inflammatory pathways. Nirmala et al. (2015) showed that most genes upregulated following a specific treatment in SoJIA patients were downregulated in the majority of AOSD patients.

The pathogenic mechanisms leading to AOSD and SoJIA are complex and not fully understood. An abnormal activation of the innate immune response is observed and several data sustain the pivotal role of inflammatory cytokines, in particular high levels of interleukin (IL)-1β, IL-6, IL-8, and tumour necrosis factor (TNF)-α (Maria et al., 2014; Sfriso et al., 2018). Thus, in addition to the classic therapeutic options (non-steroidal anti-inflammatory drugs (NSAIDs), steroids, and immunosuppressive drugs), biologic agents targeting proinflammatory cytokines may be an effective approach.

**FIGURE 1:**
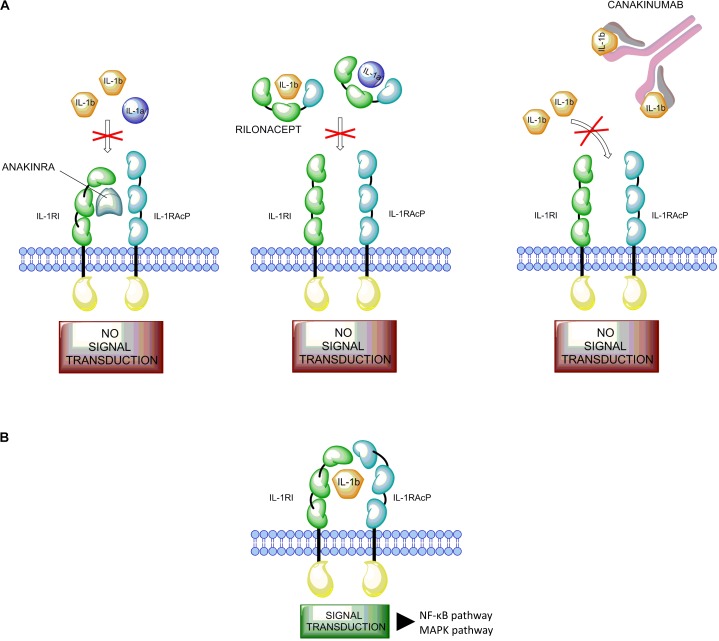
Mode of action of IL-1 and its inhibitors. **(A)** The IL-1 inhibitors (anakinra, rilonacept, and canakinumab). Anakinra (on the left) competes with free IL-1β for the binding with IL-1RI but not with the adaptor protein, thus preventing signal transduction. Rilonacept (in the middle) and Canakinumab (on the right) bind to circulating IL-1β. **(B)** Binding of IL-1β to the IL-1 receptor type I (IL-1RI) heterodimer complex results in signal transduction. IL-1β binds to the membrane-bound IL-1RI to form a complex with the IL-1 receptor accessory protein (IL-1RAcP). This complex recruits the IL-1RI-associated kinase (IRAK), leading to signal transduction and gene activation.

The IL-1 inhibitors (anakinra, rilonacept, and canakinumab; **Figure 1A**) are actively used in both AOSD and SoJIA (Giampietro and Fautrel, 2012). Anakinra is the non-glycosylated recombinant soluble antagonist of the IL-1 receptor (IL-1R) that acts preventing activation of this receptor and inhibiting both IL-1α and IL-1β activity. Rilonacept (IL-1 TRAP) is a recombinant chimeric protein composed of an extracellular domain of the human IL-1R complex fused to the Fc-portion of human IgG1. This protein is able to bind IL-1α and IL-1β with high affinity. Canakinumab is a recombinant human monoclonal antibody specifically targeting IL-1β (Astrakhantseva et al., 2014).

Previous review articles focused primarily on Anakinra, but new as well as updated data for Canakinumab have emerged with regard to the treatment not only of SoJIA but also of AOSD (Hong et al., 2014; Rossi-Semerano et al., 2015; Colafrancesco et al., 2017; Feist et al., 2018).

In this study, we summarize the initial steps of the development and the particular pharmacological characteristics of Canakinumab, reviewing currently available data on its use in the clinical setting, focusing on AOSD.

## Design and Development of Canakinumab

Among the proinflammatory cytokines, the interleukin-1 family and IL-1β in particular play a central role in immune response regulation and in the development of inflammation in Still’s disease (Dinarello, 2011). IL-1β is synthesized as inactive precursors, and the processing of pro-IL-1β is dependent on proteolytic cleavage by caspase-1, which itself is activated by a multi-protein complex called the inflammasome. A signal from IL-1 cytokine family is transduced via specific receptors (IL-1Rs) composed of an extracellular immunoglobulin-like domain that wraps around the cytokine and a Toll/IL-1 receptor (TIR) domain essential for signaling. Among the two types of IL-1Rs, type I IL-1R (IL-1RI) binds IL-1β with higher affinity than type II (IL-1RII). The complex IL-1β/IL-1RI results in a conformational change of the receptor that allows the binding with IL-1RAcP, a second receptor subunit (Wang et al., 2010; Rondeau et al., 2015). The structures of the ternary complexes of IL-1β with IL-1RAcP and IL-1RII or IL-1RI present a large binding interface between IL-1β and IL-1RI or IL-1RII, and a small surface of interaction with IL-1RAcP, facilitating interaction with receptor chains (Rondeau et al., 2015). This complex triggers a signaling cascade resulting in the activation of the NF-κB and MAPK pathways (**Figure 1B**; Auron, 1998; Dunne and O’Neill, 2003; Martin and Bugelski, 2012).

Downregulation of IL-1β activity can be achieved in two different ways. Interleukin-1 receptor antagonist (IL-1RA) can bind directly to IL-1RI preventing IL-1β and IL-1α from initiating the signal transduction or a decoy receptor, such as IL-1RII can inhibit the inflammatory response binding IL-1β, avoiding the excess of autocrine activation of the IL-1 signal.

Neutralization of the IL-1β bioactivity by an antibody targeting this cytokine could be achieved by interference with the binding of IL-1β to IL-1RI, or with the interaction between IL-1β and IL-1RAcP, or with the recruitment of IL-1RAcP into the IL-1β/IL-1RI complex (Vigers et al., 1994; Dinarello, 1998; Thomas et al., 2012; Rondeau et al., 2015). Canakinumab was generated using HuMab-Mouse TM technology from Medarex (UltiMab technology). HuMab mice are transgenic mice designed to produce fully human antibodies since their endogenous immunoglobulin repertoire is inactivated by targeted genetic disruption. These mice produce human IgG1 antibodies upon immunization with antigen, and human monoclonal antibodies are derived by conventional hybridoma technology (Fishwild et al., 1996; Lonberg, 2005; Gram, 2016). The supernatants from the hybridomas were evaluated using an ELISA screening method to determine the specificity and affinity of these antibodies, resulting ultimately in the selection of Canakinumab, a human IgG1/k antibody (Canakinumab Patent Application WO02/16436).

**FIGURE 2:**
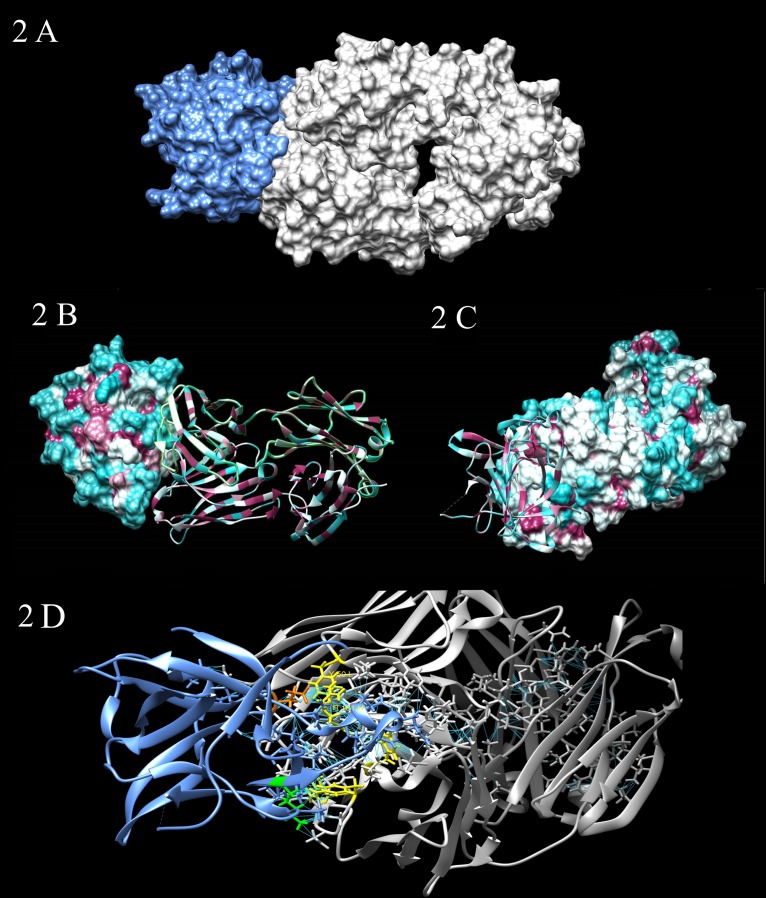
**(A)** Steric complementarity between Canakinumab and IL-1β. Canakinumab (gray) binding to IL-1β (blues) largely obeys a lock-and-key type mechanism, with contributions by all CDRs and without any large structural changes of the paratope. **(B,C)** Hydrophobic potential of Canakinumab’s Fab heavy and light chain. The surfaces are colored according to amino acid hydrophobicity. The hydrophobic residues (larger positive values of hydrophobicity) are maroon, while the hydrophilic residues (negative values of hydrophobicity) are cyan. The binding interface is remarkably flat, extensively hydrated, and very large. The IL-1β epitope does not include any aromatic or bulky hydrophobic residues. (2 B) surface epitope; (2 C) surface paratope. **(D)** All kind of interactions (polar and no polar, favorable) between Canakinumab and IL-1β. The paratope residues that stand out in terms of number of intermolecular contacts to IL-1β are colored in yellow: Arginine (Arg) H101, Tryptophan (Trp) H52, Tyrosine (Tyr) H53, Tyr H32 and Tyr L50. Arg H101 of the H-CDR3 loop plays an important role by forming strong electrostatic interactions with the epitope residue Glutamic acid (Glu) 64 (orange). Lysine (Lys) 27 (green) forms salt-bridge interactions.

The molecular formula for Canakinumab is based on the amino acid composition without post-translational glycosylation but including N-terminal pyroglutamate formation and lysine residues at the C-terminals of the heavy chains (European Medicines Agency [EMA], 2017). Both heavy chains of Canakinumab contain oligosaccharide chains linked to the protein backbone at Asparagine (Asn) 298 (Dhimolea, 2010). The X-ray structure of IL-1β/Canakinumab complex revealed that Glutamic acid (Glu) 64 in human IL-1β is a critical residue for this interaction. Canakinumab has high selectivity toward human IL-1β, assessed by competitive binding studies with soluble IL-1 receptors (Canakinumab Patent Application WO02/16436). Thus, it does not bind to other members of the IL-1 family (**Figure 2A**). Several *in vitro* studies have reported that Canakinumab interacts with human IL-1β with an equilibrium binding constant of about 40 pM and neutralizes the biological activity of IL-1β *in vivo* with an IC_50_ of about 43 pM (Alten et al., 2008; Rondeau et al., 2015).

Canakinumab also has a high degree of species specificity. It does not bind to IL-1β from macaques, rodents, canines, and many other mammalian species, since the critical residue Glu 64 is not conserved in these species (Rondeau et al., 2015). Nevertheless, the same authors identified marmosets as the only non-human primate species that carries Glu 64 in its IL-1β (like the human IL-1β) and demonstrated full cross-reactivity of Canakinumab, thereby enabling toxicological studies in this species. No toxicologically significant findings were detected (Martin and Bugelski, 2012). An embryo-fetal development study conducted in marmosets showed no major malformations but some slight skeletal variations at all dose levels of Canakinumab were suggestive of a delay in skeletal development.

Due to its high affinity and specificity for IL-1β, Canakinumab was considered suitable for therapeutic applications (Church and McDermott, 2009). The biological activity of this agent has been evaluated both *in vitro* and in animal models. The *in vitro* analysis showed a complete inhibition of IL-6 secretion stimulated by IL-1β in human dermal fibroblasts (Alten et al., 2008). Church and McDermott (2009) assessed the neutralization of IL-1β activity by Canakinumab in a mouse model of joint inflammation. The treatment proved to provide protection against severe joint destruction, with no bone erosion detected compared to controls. Demonstration of pharmacodynamic action *in vivo* was conducted in preclinical mouse model NIH 3T3. Dawson and colleagues observed that systemic intraperitoneal injections of Canakinumab in this model inhibit the neutrophil invasion in a dose-dependent manner (Gram, 2016). Mouse models for arthritis were used, instead, to validate the *in vivo* efficacy of Canakinumab. Study results demonstrated that Canakinumab can completely suppress IL-1β-mediated joint inflammation and cartilage destruction in mice (Alten et al., 2008).

In order to elucidate the molecular mechanism by which Canakinumab interferes with the IL-1β, Rondeau et al. (2015) determined the crystal structures of the Canakinumab Fab in the free and IL-1β-bound states. Canakinumab Fab subunits adopt the immunoglobulin fold and recognizes an extended, discontinuous epitope on human IL-1β. The binding interface is flat, hydrated, and very large (**Figures 2B,C**). The X-ray analysis conducted by Rondeau et al. (2015) reveals a complex surface epitope with ordered water molecules at the Canakinumab-antigen interface. These molecules contribute to the H-bonded network that connects epitope and paratope residues mediating shape/physico-chemical complementarity, improving the packing of atoms and allowing polar interactions. The amino acid composition of the paratope site is a balanced mix of hydrophobic aromatic, hydrophobic aliphatic, polar, and charged residues (Blech et al., 2013; Rondeau et al., 2015).

The IL-1β epitope forms an extended, mostly planar, and hydrophilic surface (70% of all residues are polar or charged amino-acids). The combination of electrostatic interaction and shape complementarity plays a key role in the formation of the complex between Canakinumab and IL-1β. Epitope residues engaged in direct contacts with paratope form either direct H-bonded contacts with Canakinumab or salt-bridge interactions. The epitope residues that contribute the largest number of contacts are Glu 64 that is connected to 4 CDRs, Gln 38, Lys 65, Asp 35, and Ser 258 (**Figure 2D**; Blech et al., 2013; Rondeau et al., 2015). Also, several induced-fit changes were observed at the binding interface.

The neutralizing effect of Canakinumab is caused by the direct competition for cytokine binding. There are no direct interactions between Canakinumab and IL-1RAcP. It was observed that Canakinumab and IL-1RI binding sites slightly overlap; the VH region of Canakinumab, indeed, sterically interfere with a domain of IL-1RI (Blech et al., 2013; Rondeau et al., 2015).

## Therapy

Considering the rarity of AOSD, the majority of the data on treatment management derive from empirical observations through case reports and small series, which are not standardized and may underrate the number of cases treated. Controlled clinical trials comparing the efficacies of various agents or the usefulness of different therapeutic strategies are lacking.

In order to identify the most appropriate individualized therapeutic strategy for AOSD patients, Govoni et al. (2017) recently suggested taking into consideration the following factors: the disease phase (onset, maintenance, flares), the ongoing predominant clinical features (systemic or articular) and the presence/absence of complications.

In general, the first-line treatments consist of NSAIDs and corticosteroids. In case of failure or dependence of these agents, immunosuppressive drugs such as cyclosporine A, azathioprine, leflunomide, hydroxychloroquine, methotrexate can be used (Maria et al., 2014). There is increasing evidence on the usefulness of biologic therapies in the management of corticosteroid and DMARD-refractory AOSD, reported in case series and/or national surveys (Fautrel et al., 2005; Franchini et al., 2010; Gerfaud-Valentin et al., 2014; Colafrancesco et al., 2017). The rationale for the use of biologics, actually targeting cytokines, lies in increased knowledge about the pathophysiology of the disease. Anti-TNF-α may be an attractive therapeutic option in AOSD with a predominant articular pattern (Gerfaud-Valentin et al., 2014), while IL-1β inhibitors are more effective for the systemic manifestations (Vitale et al., 2016). The use of an IL-6 antagonist has proved to be useful for both joint and systemic manifestations (Cipriani et al., 2014).

There are also no guidelines for decisions about how long to continue biologics, once clinical remission is achieved. In patients with systemic AOSD, response to IL-1 inhibitors must be expected within hours or days; in the articular subset, the response to anti IL-6 or anti-TNF may require more time, but the low disease activity should be achieved within no more than 3 months. If clinical remission is maintained for at least 6–12 months, a cautious attempt to taper the biologic could be pursued (Govoni et al., 2017).

Like in SoJIA and other autoinflammatory diseases, targeting IL-1β seems particularly relevant in AOSD, in agreement with the several case series and national surveys on it (see Junge et al., 2017 for a comprehensive review).

Canakinumab is indicated for the treatment of different autoinflammatory syndromes in adults, adolescents and children aged 2 years and older. In 2009, it was approved by the United States Food and Drug Administration (FDA) for the treatment of cryopyrin-associated periodic syndromes (CAPS) and active systemic juvenile idiopathic arthritis (SoJIA). In 2013, the European Commission approved Canakinumab for adult patients with frequent gouty arthritis attacks. In 2016, the FDA approved expanded indications for tumour necrosis factor receptor associated periodic syndrome (TRAPS), mevalonate kinase deficiency (MKD), familial Mediterranean fever (FMF) in combination with colchicine. In 2016, the EMA also approved Canakinumab for a license extension to treat patients with Adult-onset Still’s disease (AOSD), supported by the concept of a Still’s disease continuum that includes both juvenile and adult onset forms (Nirmala et al., 2015). The population-based pharmacokinetics-binding model of Still’s disease by Feist et al. (2018) showed that Canakinumab exposure is comparable across all ages.

Sun et al. (2016) characterized the pharmacokinetic (PK) and pharmacodynamic (PD) properties of Canakinumab in SoJIA patients aged under 20, to evaluate adequacy of exposure of the drug and the recommended dosage in the population. A population-based PK-binding model was built to evaluate Canakinumab PK properties and total IL-1β kinetic properties. The model showed that Canakinumab clearance and volume of distribution were not impacted by age after correction for the patient’s body weight. Canakinumab exhibited dose-proportional exposure with increasing doses in patients with SoJIA, as expected. The pharmacokinetic modeling analysis also predicted Canakinumab steady state after 110 day exposures at a dose level of 4 mg/kg in SoJIA patients. These data would be comparable to those in older SoJIA patients, who represent AOSD patients.

The recommended posology for patients with Still’s disease (AOSD and SoJIA) with body weight ≥7.5 kg is 4 mg/kg (up to a maximum of 300 mg) administered every 4 weeks via subcutaneous injection (European Medicines Agency [EMA], 2017). Up to now, the use of Canakinumab has been reported in 24 AOSD patients (Kontzias and Efthimiou, 2012; Banse et al., 2013; Eriksson et al., 2013; Barsotti et al., 2014; Lo Gullo et al., 2014; Maria et al., 2014; Rossi-Semerano et al., 2015; Colafrancesco et al., 2017; Sinha et al., 2018) and 29 older adolescents/young adults representing AOSD patients (Feist et al., 2018), refractory to NSAIDs, DMARDs and often to other biologic therapy. The majority of patients showed a rapid and sustained efficacy with subcutaneous injection of 150 mg every 8 weeks. All the reports and their results are summarized in **Table 1**. Canakinumab was reported effective in treating arthritic symptoms as well as systemic symptoms in 9 out of 14 AOSD patients (Kontzias and Efthimiou, 2012; Banse et al., 2013; Eriksson et al., 2013; Barsotti et al., 2014; Maria et al., 2014; Rossi-Semerano et al., 2015; Colafrancesco et al., 2017). Of note, Lo Gullo et al. (2014) reported a refractory case of AOSD in which Canakinumab was efficient only for systemic manifestations, while joint involvement remained active with high disease activity scores. Moreover, the pooled analysis from SoJIA studies by Feist et al. (2018) indicates that consistent clinical benefits, represented by clinical and inflammatory laboratory markers and quality of life (QoL) measures, observed in older adolescents and young adults are comparable to those seen in children and young adolescents.

**Table 1:** Reports of Canakinumab use in AOSD patients.

Reports	# Patients and dosage	Results
Kontzias and Efthimiou, 2012	n. 2 patients with refractory AOSD received 150 mg of Canakinumab s.c. injections every 8 weeks	– Resolved systemic symptoms, – normalized laboratory values, – no AEs (except transient diarrhea in 1 of 2 patients), – no relapse in 6–12 months
Banse et al., 2013	n. 1 patient with refractory AOSD received two doses of 150 mg of Canakinumab s.c. injections per week	– Development of MAS after the second injection at 3 and 6 months after MAS, – resolved symptoms and normalized laboratory values
Eriksson et al., 2013	n. 1 patient with refractory AOSD received 150 mg of Canakinumab s.c. injections every 8 weeks	– Resolved joint and skin symptoms – normalized laboratory values
Barsotti et al., 2014	n. 1 patient with refractory AOSD received 150 mg of Canakinumab s.c. injections every 4 weeks, gradually increasing up to 150 mg every 8 weeks	– Three brief episodes of fever, not temporally related to Canakinumab – no systemic and joint symptoms at 18 month follow-up
Lo Gullo et al., 2014	n. 1 patient with refractory AOSD received 150 mg of Canakinumab s.c. injections every 8 weeks	– No systemic symptoms at 14 month follow-up – normalized laboratory values – recurrent arthritis required methylprednisolone 12 mg/day
Maria et al., 2014	n. 1 patient with refractory AOSD received Canakinumab (dosage not reported)	– Rapid and complete remission of systemic symptoms at 30 month follow-up
Rossi-Semerano et al., 2015	A French nationwide survey includes n. 2 patients with refractory AOSD receiving 150 mg of Canakinumab s.c. injections every 4 and 8 weeks, respectively	– Complete remission in 1 patient receiving 150 mg/8 week – no clinical improvement in 1 patient receiving 150 mg/4 week
Colafrancesco et al., 2017	An Italian nationwide survey includes n. 4 patients with refractory AOSD receiving 150 mg of Canakinumab s.c. injections every 8 weeks	– Normalized laboratory values – effective in 3 out 4 patients – loss of efficacy in 1 patient with chronic articular AOSD – no AEs
Feist et al., 2018	Pooled analyses from SoJIA studies (NCT00426218, NCT00886769, NCT00889863, and NCT00891046) include n. 29 older adolescents/young adults (representing AOSD patients), receiving 4 mg/kg every 4 weeks	– At day 15, 19 out 29 older adolescents/young adults have aACR ≥ 70 responses and showed improvements (13 out 18) at day 85. – Normalized laboratory values
Sinha et al., 2018	n. 1 patient with refractory AOSD and severe pulmonary hypertension received Canakinumab (dosage not reported)	– No clinical improvement
Trial NCT02204293 (Fase II)	A 12-week trial including 68 AOSD patients randomized into two groups receiving either placebo or 150 mg of Canakinumab s.c. injections every 8 weeks. The aim of the study is to assess the efficacy, safety, and tolerability of Canakinumab	– The primary outcome measures will be available in June 2019

*s.c., sub-cutaneous; AOSD, Adult-onset Still’s disease; AEs, adverse events; MAS, macrophage activation syndrome; SoJIA, systemic onset juvenile idiopathic arthritis; aACR, adapted American College of Rheumatology.*

In the listed reports (**Table 1**), Canakinumab was effective in the most difficult-to-treat cases of refractory AOSD, except in two patients (Rossi-Semerano et al., 2015; Sinha et al., 2018). The former was a refractory AOSD patient with a rare cardiopulmonary manifestation (severe pulmonary hypertension), the latter was a refractory AOSD patient receiving 150 mg/4 week Canakinumab.

Overall, Canakinumab was well-tolerated and only two adverse events (AEs) were reported (Kontzias and Efthimiou, 2012; Banse et al., 2013). One is a MAS, a serious AE suggested by Banse et al. (2013) to be related to the drug, even though MAS is a frequent complication of AOSD. The other is a transient diarrhea in a good responder patient.

Interactions between Canakinumab and other drugs have not been investigated in formal studies. An increased safety risk and raised incidence of serious infections and injection-site reactions have been associated with combination therapy of Anakinra and TNF inhibitors in the treatment of patients with rheumatoid arthritis (Genovese et al., 2004). Thus, the use of Canakinumab with TNF inhibitors is not recommended because this may increase the risk of serious infections.

Canakinumab has a well-established safety and efficacy profile in SoJIA, as reported by recently published SoJIA clinical trials (NCT00889863, NCT00886769, NCT00891046, and NCT00426218) (Ruperto et al., 2012a,b; Sun et al., 2016). Clinical endpoints such as ACR criteria, including adapted American College of Rheumatology pediatric criteria (aACR) responses, improved rapidly; at day 15, indeed, at least 50% of patients, aged between 2 and 20 years, had aACR ≥ 70 responses and were maintained or improved over 12 weeks of treatment. Interestingly, the pooled analysis from SoJIA patients indicates that older adolescents exhibit similar efficacy, safety, and exposure-response relationship on a weight-based dosing regimen to children and adolescent SoJIA patients (Feist et al., 2018).

Concerning AOSD, a multicentre, placebo-controlled clinical trial (NCT02204293) is currently ongoing. The aim of this interventional study is to investigate the efficacy of Canakinumab in 68 patients with AOSD and active joint involvement in terms of the proportion of patients with a clinically significant reduction in disease activity (DAS28 > 1.2) following a treatment period of 12 weeks.

Consistent with prior reports (**Table 1**), data on complete or partial remission in most patients treated with Canakinumab also emphasized the efficacy of this drug in AOSD. The initial efficacy of Canakinumab seems not to be clearly different from that of the other two IL-1 inhibitors. Both Anakinra and Rilonacept indeed showed rapid and sustained efficacy (Henderson et al., 2010; Hong et al., 2014), but it can be lost over time, reflecting their shorter half-life (4–6 h and 9 days, respectively) compared to that of Canakinumab (26 days) (Kontzias and Efthimiou, 2012).

Canakinumab is also better tolerated than the other IL-1 inhibitors (Henderson et al., 2010; Rossi-Semerano et al., 2015). Beyond the common side-effects of Anakinra and Rilonacept (injection site reactions), both agents presented few severe AEs.

Taken together, this substantial evidence endorses the use of IL-1 inhibitors in AOSD patients refractory to other treatments, even as early treatments (Pouchot and Arlet, 2012). Their ability to interrupt the disease process suggested the possibility of shortening flares and avoiding chronic relapses.

## Author Contributions

PS and FO conceived and designed the study. PG, CB, and SB drafted the manuscript. All authors have critically reviewed the draft manuscript.

## Conflict of Interest Statement

The authors declare that the research was conducted in the absence of any commercial or financial relationships that could be construed as a potential conflict of interest. The reviewer AC and handling Editor declared their shared affiliation at time of review.
